# Bacterial dynamics in the progression of caries to apical periodontitis in primary teeth of children with severe early childhood caries

**DOI:** 10.3389/fmicb.2024.1418261

**Published:** 2024-09-11

**Authors:** Bichen Lin, Jinfeng Wang, Yifei Zhang

**Affiliations:** ^1^First Clinical Division, Peking University School and Hospital of Stomatology and National Center of Stomatology and National Clinical Research Center for Oral Diseases and National Engineering Research Center of Oral Biomaterials and Digital Medical Devices, Beijing, China; ^2^College of Food Science and Nutritional Engineering, China Agricultural University, Beijing, China; ^3^Department of Dental Materials, Peking University School and Hospital of Stomatology and National Center for Stomatology and National Clinical Research Center for Oral Diseases and National Engineering Research Center of Oral Biomaterials and Digital Medical Devices, Beijing, China

**Keywords:** severe early childhood caries, apical periodontitis, bacteria of different layers, co-occurrence network, 16S rRNA sequencing

## Abstract

**Background:**

Early childhood caries (ECC) are a prevalent chronic disease in young children. However, there has been limited research on the microbiota in different tissue levels of the same tooth in children with ECC. This study aimed to investigate the dynamic changes in bacterial diversity during the progression of Severe Early Childhood Caries (S-ECC) within the same tooth, from the tooth surface to the root canal, by collecting tissue samples from different areas of the affected tooth.

**Methods:**

Twenty primary teeth with periapical periodontitis were selected from 20 children aged 3–5 years, with 100 samples collected from the different layers: uncavitated buccal enamel surface without white spot lesion (surface), the outermost layer of the dentin carious lesion (superficial), the inner layer of carious dentin (deep), necrotic pulp tissue (pulp), and root exudate (exudate). The taxonomy of each OTU representative sequence was analyzed against the 16S rRNA database. Comparisons of alpha diversity between groups were performed. The number of shared and unique genera between groups counted. Beta diversity was contrasted to evaluate differences in bacterial community composition, and the relationships between the microbiota and samples were analyzed. The heatmap analysis of the 30 most abundant genera was used, which highlighted their relative distribution and abundance. The significantly abundant taxa (phylum to genera) of bacteria among the different groups were identified. The differences of relative abundance between bacterial genera among the five groups were analyzed. Significant Spearman correlations were noted, and visualization of the co-occurrence network was conducted.

**Results:**

Bacterial 16S rRNA gene sequencing showed that most genera were present in all layers, with the number of shared genera increasing as the disease advanced. The bacterial communities and core genera in the co-occurrence network changed with progression to severe ECC.

**Conclusion:**

An increase in both the quantity and complexity of bacterial interactions was observed. This study emphasized the importance of paying attention to the relationship between microbial species rather than just checking changes in bacterial species structure when investigating the role of bacteria in disease progression.

## 1 Introduction

Early childhood caries (ECC) is a prevalent chronic disease in young children, affecting as many as 72.7% of impoverished preschoolers in both developing and industrialized countries (Dye et al., 2015). The terms early childhood caries (ECC) and severe ECC (S-ECC) were first introduced in the 1990s (Ismail and Sohn, 1999). ECC is defined as the presence of one or more cavities, missing teeth (due to decay), or filled tooth surfaces in children aged 72 months or younger (American Academy of Pediatric Dentistry, 2016). S-ECC is the severe form of ECC and has an important effect on children’s development and well-being (Pierce et al., 2019; Folayan et al., 2020). Severe early childhood caries (S-ECC) is characterized by the presence of one or more cavitated, missing teeth (due to decay), filled smooth surfaces in primary maxillary anterior teeth, or a decayed, missing, or filled surface score of ≥4 (age 3), ≥5 (age 4), or ≥6 (age 5) (American Academy of Pediatric Dentistry, 2016). S-ECC progresses rapidly and aggressively, causing pain and requiring frequent and costly oral rehabilitation under general anesthesia (Hajishengallis et al., 2017). If left untreated, ECC quickly leads to pulpitis and apical periodontitis, potentially resulting in dento-alveolar abscesses and the spread of infection to other areas of the body. S-ECC therefore presents a significant challenge for public health systems (Dye et al., 2007).

The formation of plaque biofilm, a buildup of oral microbes on the tooth surface is the primary cause of dental caries. Numerous studies have identified a specific group of acid-producing and acid-resistant bacteria, particularly *Streptococcus mutans*, as the main culprit in dental plaque (Richards et al., 2017; Xiao et al., 2018a). An infected root canal of primary teeth contains a diverse range of bacteria, including both obligate and facultative anaerobic species, with necrotic pulp and apical periodontitis teeth containing a significant number of anaerobic bacteria (Ruviére et al., 2007; Siqueira et al., 2009). It is important to note that caries, pulpitic, and apical periodontal disease are all stages of the same disease, with caries being the initial stage. However, previous studies on ECC have often analyzed these different lesions separately, rather than considering them as interconnected stages of the same disease.

During the process of caries development into apical periodontitis, the microenvironment of the lesion undergoes continuous changes during disease progression. As a result, the structure and function of the microbiota also adapt, making it important to identify which microbes play a significant role in different stages of the lesion and understand the characteristics of their interaction. Although this knowledge is crucial for understanding the development and progression of the disease, these issues have not been studied thoroughly, with a focus on a single type of lesion that possibly has resulted in important information being missed. Previous studies have mostly limited the collection of samples at a single or two-layer depth of caries, and there has also been limited research on the microbiota in different levels of the same tooth in children with ECC.

To address this gap, this study aimed to investigate the dynamic changes in bacterial diversity during the progression of Severe Early Childhood Caries (S-ECC) within the same tooth, from the tooth surface to the root canal, by collecting tissue samples from different areas of the affected tooth. We hypothesized that while the species of bacteria may not have varied significantly at different depths, the bacterial diversity and interactions did undergo changes.

## 2 Materials and methods

### 2.1 Study subjects and sample collection

This study was approved by the Ethical Committee of Peking University School and Hospital of Stomatology (Beijing, China) (approval number: PKUSSIRB-202281143). Written, informed consent was obtained from the guardians of all participants prior to recruitment.

Children diagnosed with S-ECC after clinical assessment of the primary teeth and who needed dental treatment under general anesthesia with nasotracheal intubation were enrolled in the study. The diagnoses were made by an experienced pediatric dentist at the department of pediatric dentistry in Peking University Hospital of Stomatology First Clinical Division, according to the WHO oral health survey basic methods (5th edition). The diagnosis of S-ECC consisted of three major parts: (1) any sign of smooth-surface caries in a child <3-years-old, (2) the presence of one or more decayed, missing (due to caries), or filled smooth surfaces in any primary maxillary anterior teeth in children aged 3–5 years, and (3) DMFT (decayed, missing, filled tooth) index ≥4 (age 3), ≥5 (age 4), or ≥6 (age 5) (American Academy of Pediatric Dentistry, 2016). The exclusion criteria were: (1) received antibiotics or fluoride treatment during the past 3 months, and (2) suffered from other oral infections or systematic diseases. Clinical samples were taken from the teeth with pulp necrosis and apical periodontitis that were diagnosed by clinical (presence of tissue swelling, percussion sensitivity, fistula, symptomatic or asymptomatic) and periapical radiographs with a clear periapical radiolucency. The occlusal caries was deep like pulpal quarter but still with radiopaque dentine in between in radiographs (Duncan et al., 2019). All participants had at least one primary tooth with apical periodontitis.

**Figure 1:**
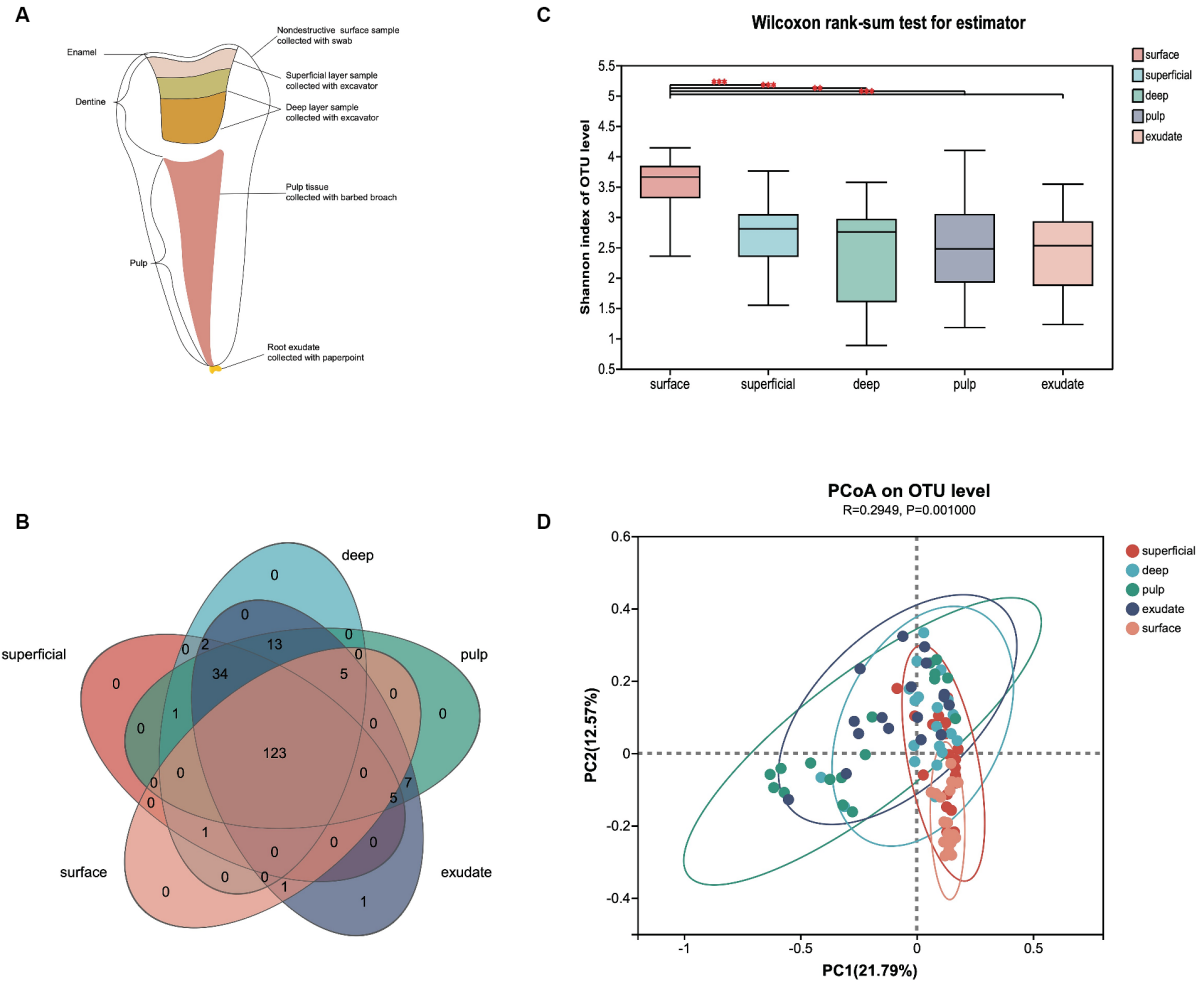
**(A)** Schematic diagram of each sample lesion defined in the study. **(B)** Venn diagram of exclusive and shared bacterial OTUs (3% evolutionary distance) in the different sample groups. **(C)** The Shannon index of the bacterial community from surface to exudate. Horizontal lines represent mean values. *p* < 0.05 indicates statistical significance in the Wilcoxon rank-sum test. **(D)** Principal coordinates analysis (PCoA) on unweighted UniFrac distances in the five groups, shown along the first two principal coordinate (PC) axes.

All the participants were instructed to not brush their teeth on the morning of sampling and to abstain from eating or drinking for at least 4 h prior. Following local anesthesia, a rubber dam was used to isolate the carious tooth from saliva contamination. No rubber dam leakage was observed during the sampling procedures. The plaque from uncavitated buccal enamel surface without white spot lesion (surface) was collected using a sterile cotton swab. The decayed occlusal enamel was first removed with a water-cooled diamond bur handpiece operated at low speed. The outermost layer of the dentin carious lesion (superficial) was rinsed with a sterile saline solution, and a bacteria-containing superficial layer of the carious dentin lesion was collected using a sterile spoon excavator. After rinsing with sterile saline, a similar amount of the inner layer of carious dentin (deep) was collected with a new excavator from the same carious lesion (Liu et al., 2020). No pulp tissue was exposed during caries sample collection. Before the pulp chamber was exposed, the cleaning of the tooth and rubber dam was repeated as previously described. After gaining entrance to the root canal system, the necrotic pulp tissue (pulp) was collected using Mani barbed broaches (Mani Inc., Tochigi, Japan), with a sterile paper point used to absorb any exudate (exudate) in the canal (Gomes et al., 2008; Santos et al., 2011; Hong et al., 2013) (Figure 1A). Each sample was placed in a 1.5 mL centrifuge tube containing 50 μL of sterile phosphate buffer saline (pH = 7.2, Hyclone, United States) and stored immediately at −80°C.

This study included 33 primary molars from 33 children diagnosed with severe early childhood caries (S-ECC), resulting in a total of 166 samples collected. Samples exhibiting pulp necrosis were identified through screening, while those with potential contamination risk were excluded from the analysis. At last 20 primary teeth with periapical periodontitis from 20 children (aged 3–5 yrs) were selected and 100 samples from the different layers: uncavitated buccal enamel surface without white spot lesion (surface), the outermost layer of the dentin carious lesion (superficial), the inner layer of carious dentin (deep), necrotic pulp tissue (pulp) and root exudate (exudate) were included in the analyses. The mean DMFT index for these children was 14.40 (±2.66), indicating a high prevalence of dental caries. Additionally, the average number of teeth affected by apical periodontitis per child was 3.85 (±2.43).

### 2.2 DNA isolation and 16S amplicon sequencing

The samples were pre-treated with lysozyme for better cell wall lysis and genomic DNA was extracted from each sample using the E.Z.N.A.^®^ soil DNA Kit (Omega Bio-tek, Norcross, GA, United States) according to the manufacturer’s instructions. The hypervariable region V3-V4 of the bacterial 16S rRNA gene was amplified with primer pairs 338F (5′-ACTCCTACGGGAGGCAGCAG-3′) and 806R (5′-GGACTACHVGGGTWTCTAAT-3′). Purified amplicons were pooled in equimolar and paired-end sequences on an Illumina MiSeq PE300 platform/NovaSeq PE250 platform (Illumina, San Diego, United States) by Majorbio Bio-Pharm Technology Co. Ltd. (Shanghai, China). The raw reads were deposited into the NCBI Sequence Read Archive (SRA) database (Accession Number: PRJNA1129318).

### 2.3 Data processing

The raw 16S rRNA gene sequencing reads were demultiplexed, quality-filtered by fastp version 0.20.0 (Chen et al., 2018) and merged by FLASH version 1.2.11 (Magoč and Salzberg, 2011) according to the following criteria: (i) the 300 bp reads were truncated at any site receiving an average quality score of <20 over a 50 bp sliding window, while truncated reads shorter than 50 bp and those containing ambiguous characters were discarded; (ii) only overlapping sequences longer than 10 bp were assembled according to their overlapped sequence. The maximum mismatch ratio of the overlap region was 0.2. Reads that could not be assembled were discarded; (iii) Samples were identified by the barcode and primers, with the sequence direction adjusted to match the exact barcode of two nucleotide mismatches in primer matching.

### 2.4 Data analyses

Operational taxonomic units (OTUs) with a 97% similarity cutoff were clustered using UPARSE version 11 (Edgar, 2013), and chimeric sequences then identified and removed. The taxonomy of each OTU representative sequence was analyzed by RDP Classifier version 2.13 (Wang et al., 2007) against the 16S rRNA database (Silva v138) using a confidence threshold of 0.7. The alpha diversity was computed using Shannon index (richness and evenness) with Mothur version 1.30.2 (Schloss et al., 2009). and the beta diversity by an un-weighted Unifrac metric. Comparisons of alpha diversity between groups were performed using the Wilcoxon rank-sum test. A Venn diagram was used to count the number of shared and unique genera between groups. Beta diversity was visualized by principal coordinate analysis (PCoA) to evaluate differences in bacterial community composition based on unweighted UniFrac metrics, while a Circos graph was used to visualize the relationships between the microbiota and samples. The relative abundance on the genus level was showed with community barplot analysis. To investigate differences in the bacterial communities of the five groups, we used heatmap analysis of the 30 most abundant genera, which highlighted their relative distribution and abundance. The linear discriminant analysis (LDA) effect size (LEfSe)^1^ was performed to identify the significantly abundant taxa (phylum to genera) of bacteria among the different groups (LDA score > 2, *p* < 0.05) (Segata et al., 2011). Mann–Whitney test with false discovery rate (FDR) correction was used to analyze the differences of relative abundance between bacterial genera among the five groups. Significant Spearman correlations (*p* < 0.01, *R* ≥ 0.5) were noted, and visualization of the co-occurrence network was conducted. Possible keystone genera were those that demonstrated high betweenness centrality values (Vick-Majors et al., 2014; Xue et al., 2017). The modular structure of the community was evaluated via the modularity index (Damseh et al., 2021). Statistical analyses were performed using the SPSS software, version 26.0 (SPSS Inc., Chicago, IL, United States). A *p* value < 0.05 was considered statistically significant.

## 3 Results

### 3.1 Sample collection and data description

After sample processing and data quality filtering of the 100 samples, 93 were available for further analysis. A total of 5,674,423 high-quality bacterial V3-V4 region sequence reads were obtained, with an average of 61,015 per sample. The filtered sequence reads were clustered into operational taxonomic units (OTUs), and classified to taxa using the Silva database (Release138).^2^ The 525 OTUs identified were assigned to 16 phyla, 24 classes, 57 orders, 101 families, and 193 genera at a 3% evolutionary distance. A Venn diagram showed that 123 genera were shared among all five sample groups, with the number of shared genera increasing as the disease progressed (Figure 1B). Only the exudate group had one site-specific genus, while the surface group shared the least genera with the other groups.

### 3.2 Bacterial diversity

To describe the changes in different infectious layers, we first compared the α-diversity of bacterial communities in the groups. The variations in microflora from surface to exudate are shown in Figure 1C. Samples from the surface had significantly higher α-diversity than the other regions (superficial, *p* < 0.000; deep, *p* < 0.000; pulp, *p* < 0.004; exudate, *p* < 0.001), while there was no significant difference in the Shannon index from superficial to exudate. Principal coordinate analysis (PCoA) based on un-weighted UniFrac distance showed that the community structure of the surface was more similar to the superficial infections, while deep, pulp, and exudate were close to each other (*p* = 0.001), with a R2 value of 0.29 suggesting lesion site variation. Interestingly, the bacterial communities shifted progressively with the progression of S-ECC from the early caries stage to the late periapical periodontitis stage (Figure 1D).

### 3.3 Bacterial community profiles

**Figure 2:**
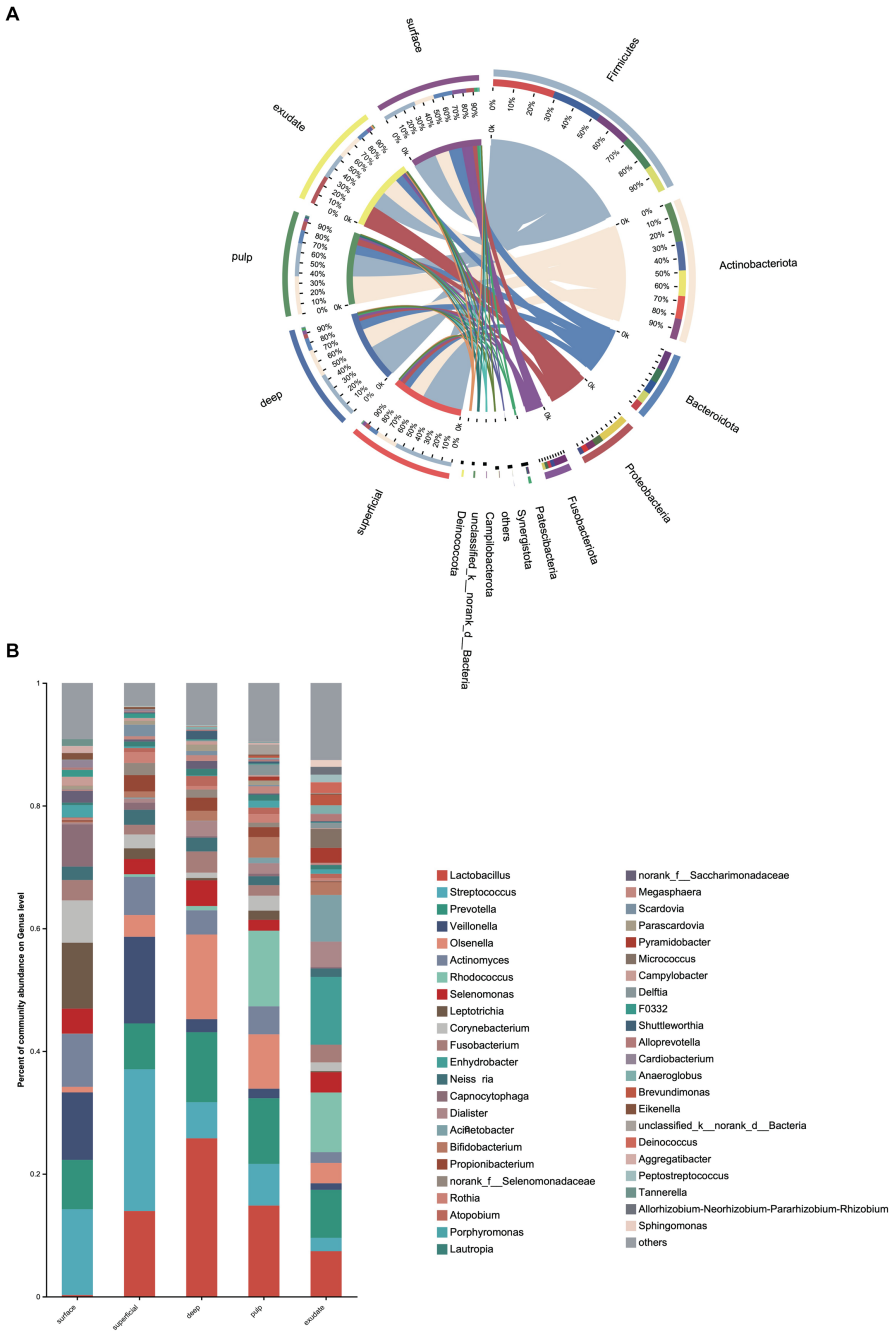
**(A)** Heat map of genus levels in the samples. The sample groups are identified by different colors. Each row represents a genus and each column represents an individual sample. The density of the color in each cell represents the count of the taxon in that sample. **(B)** Linear discriminant analysis (LDA) effect size taxonomic cladogram showing differentially abundant taxa in each group.

**Figure 3:**
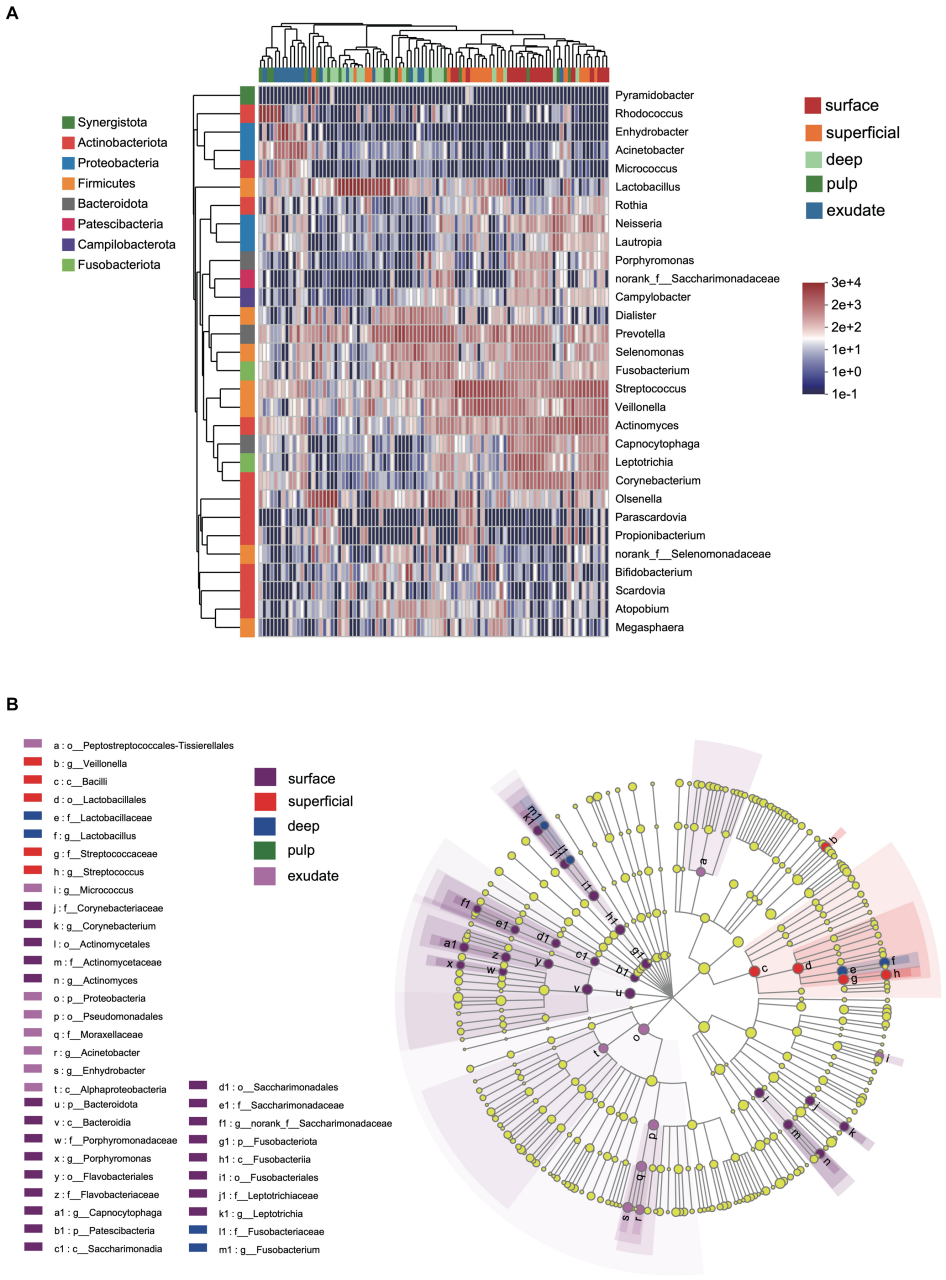
Bacterial genera with significant (Mann–Whitney test with false discovery rate (FDR) correction; *p < 0.05) differences among the five groups.

We next investigated differences in the taxonomic composition between the groups. At the phylum level, Firmicutes, Actinobacteria, Bacteroidota, Proteobacteria, and Fusobacteria were the most abundant taxa in all the groups. The superficial group had a higher proportion of Firmicutes, while the exudate group had a higher proportion of Proteobacteria. The relative abundance of Firmicutes decreased gradually from superficial to exudate, Proteobacteria increased gradually from deep, pulp, to exudate, while Actinobacteria increased from surface, superficial, deep, to pulp (Figure 2A). At the genus level, 53 prevalent genera with >1% average relative abundance and at least 1% relative abundance in more than five samples were observed. The surface samples were dominated by *Streptococcus*, *Prevotella*, *Veillonella*, *Actinomyces*, and *Leptotrichia*. In the superficial dentin group, *Streptococcus*, *Lactobacillus*, and *Olsenella* were increased compared with the surface group, while the deep dentin layer contained a much higher proportion of *Lactobacillus* and *Olsenella* but lower *Streptococcus* and *Veillonella*. *Streptococcus*, *Veillobella,* and *Actinomyces* were no longer dominant in the necrotic pulp and exudate samples, with *Rhodococcus* constituting a significant part of the communities, while *Enhydrobacter* and *Acinetobacter* were only dominant in the exudate group (Figure 2B). A heatmap generated by cluster analysis showed formation of several subgroups of taxonomic profiles, with one subgroup including mainly exudate samples and other surface samples. As shown in Figure 3A, the samples from the surface and superficial regions and from the deep, necrotic pulp, and exudate regions could be grouped together.

**Figure 4:**
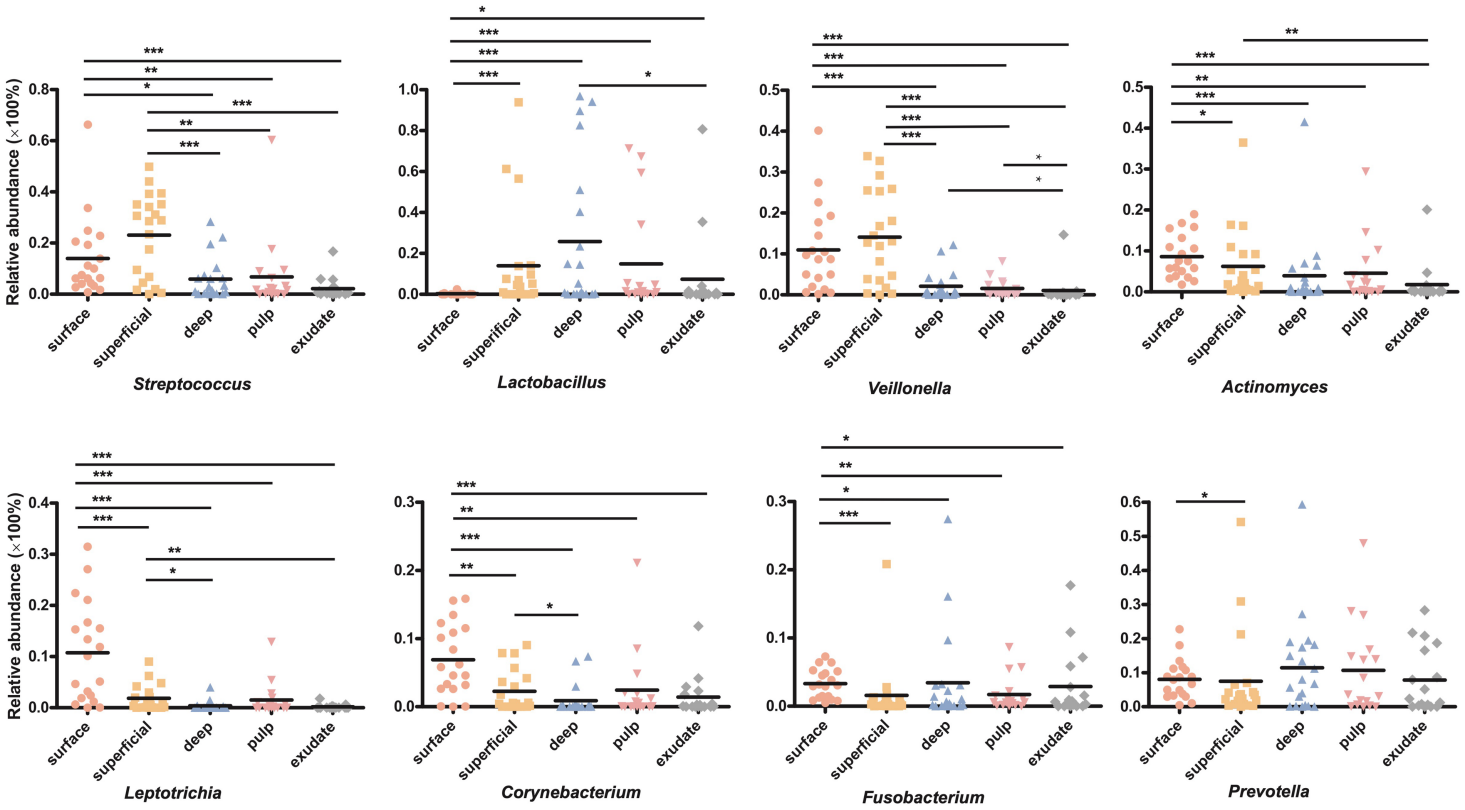
**(A)** Circos graph of the composition of the phylum levels in each group. **(B)** Relative abundance of the top 50 annotated microbial taxa at the genus level.

LEfSe analysis showed 38 discriminating taxon features among the five groups across different taxonomic levels (Figure 3B). There were more genera with significant differences on the tooth surface, followed by the decayed dentin, with the lowest significant differences found in the necrotic pulp and exudate. At the genus level, the prevalent bacteria included *Corynebacterium*, *Actinomyces*, *Porphyromonas*, *Capnocytophaga,* and *Leptotrichia* in the surface samples, *Streptococcus* and *Veillonella* in superficial samples, and *Lactobacillus* and *Fusobacterium* in deep samples. *Micrococcus*, *Acinetobacter*, and *Enhydrobacter* were only prevalent in exudate samples. All these biomarkers had high LDA scores (>4), which reflected the statistically and biologically significant differences in abundance of these microbial communities. Study of the changes in the relative abundance of key genera from the surface to the root canal exudate showed differences for *Streptococcus*, *Veillonella*, *Leptotrichia*, and *Corynebacterium*. Relative to surface samples, *Streptococcus* was diminished significantly in the deep, necrotic pulp, and exudate samples, *Lactobacillus* was most prevalent in superficial, deep, and necrotic pulp samples, *Veillonella* was enriched in superficial but diminished in deep, necrotic pulp, and exudate samples, *Actinomyces* decreased gradually from the surface, superficial, to exudate samples, *Fusobacterium*, *Leptotrichia*, and *Corynebacterium* were diminished in all groups, whereas *Prevotella* was only slightly diminished in the superficial samples (Figure 4).

### 3.4 Changes in the bacterial community co-occurrence network with S-ECC progression

**Figure 5:**
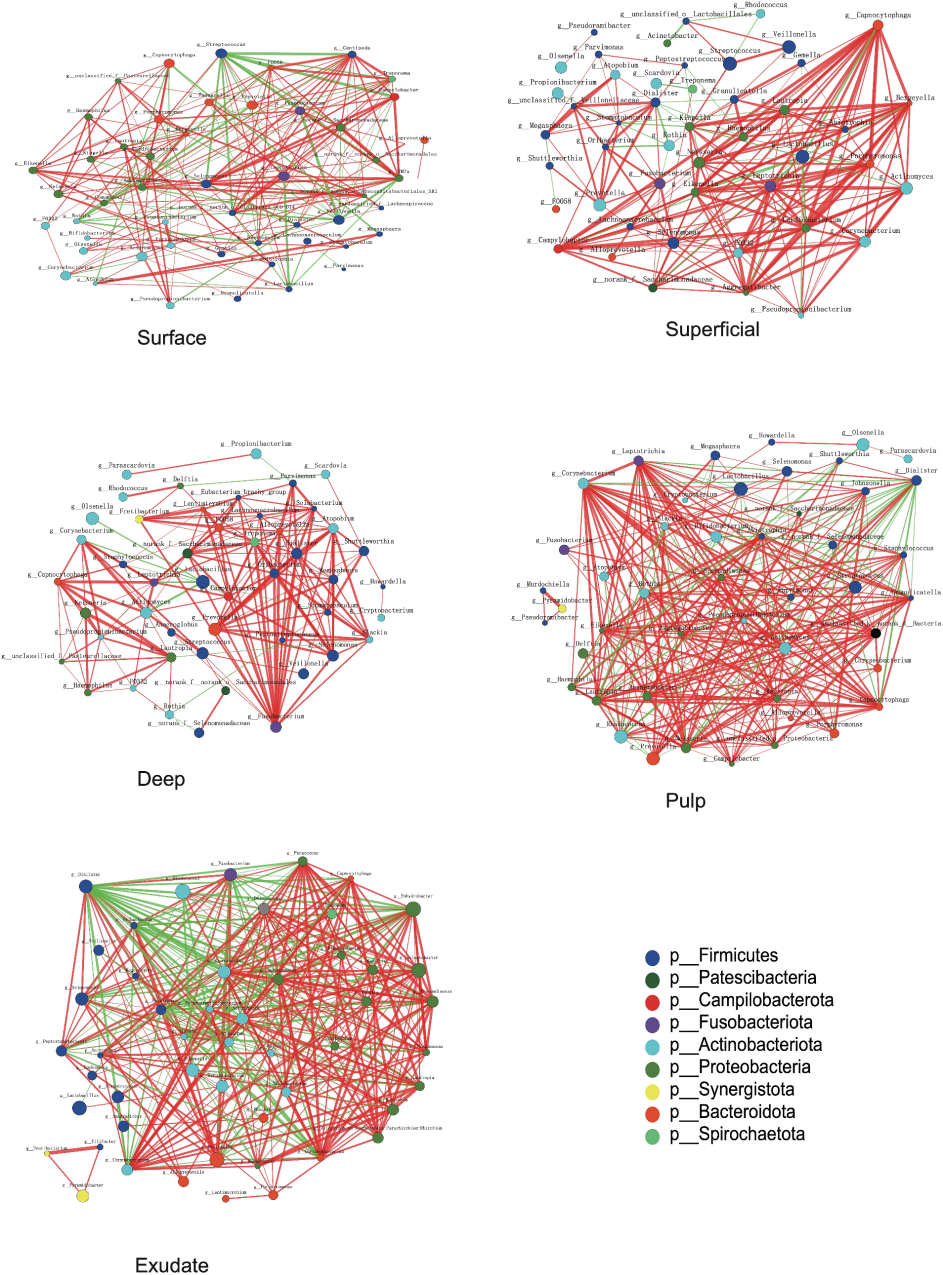
Bacterial co-occurrence networks of surface, superficial, deep, pulp, and exudate groups. Each microbial genus and cooccurrence relationship are indicated by a node and an edge, respectively. The graph’s node size reflects the abundance of genus, while different colors indicate different phyla. Lines between nodes indicate positive correlations (red) or negative correlations (green). The thickness of the lines corresponds to the correlation coefficient’s magnitude, with thicker lines indicating a stronger correlation between genera. Additionally, the number of lines indicates the strength of the connection between a particular genus and others.

The interaction patterns between bacteria in different stages of S-ECC were analyzed by constructing co-occurrence networks among the most related genera based on Spearman correlation. Approximately 41 ~ 44 genera (nodes) and 190 ~ 346 connections (edges) were retained using different correlation cut-offs (*R* ≥ 0.5, Figure 5). We calculated topological features including betweenness centrality (number of shortest paths through a node), closeness centrality (number of steps required to access all other nodes from a given node), and degree centrality (number of adjacent edges). Based on these scores, the main genera in the different groups were *Centipeda, Treponema, TM7x, Veillonella*, and *Streptococcus* in the surface group*, Kingella, Capnocytophaga, Porphyromonas, Eikenella and Neisseria* in superficial lesions, and *Fusobacterium, Treponema, Dialister, norank_f__Saccharimonadaceae and Alloprevotella* in deep lesions. The most prevalent genera in necrotic pulp samples were*_Corynebacterium, Eikenella, Leptotrichia, Lautropia*, and *Ralstonia*, and in exudates, *Dialister, Corynebacterium, Sphingomonas, Lautropia*, and *Parvimonas.* Most of the interactions were classified as positive in the constructed networks (i.e., 156, 177, 178, 238, and 234 positive (75, 84.3, 93.7, 82.4, 67.8%) and 52, 33, 12, 51, 112 negative (25, 15.7, 6.3, 17.6, 32.2%) for interactions in surface, superficial dentin, deep dentin, necrotic pulp and exudate samples, respectively).

Most of the relationships between *Streptococcus* and other bacterial genera in the superficial plaque bacterial interactions network were negatively correlated (81.25%), with the majority of these relationships being statistically significant (76.9%, *p* < 0.01). However, there was a positive correlation between *Lactobacillus* and *Streptococcus* (*p* < 0.01). Notably, *Lactobacillus* had negative relationships with other bacteria in the superficial and deep dentin caries layers.

In superficial and deep dentin caries, *Capnocytophaga* and *Fusobacterium* were at the core of the network, respectively. Most interactions between *Capnocytophaga* and other genera showed positive correlations (19/20), with most being statistically significant (*p* < 0.01, 16/20), while *Fusobacterium* interacted positively with all other genera (20/20), with most correlating significantly (*p* < 0.01, 18/20).

In the superficial and deep layers of caries lesions, there were more interactions between bacteria than on the tooth surface, stronger positive correlations than on the tooth surfaces (surface, 75.0%; superficial layer, 84.2%; deep layer, 93.7%), and more significant correlations (surface, 53.4%; superficial layer, 71.9%; deep layer, 70.5%). The interactions between the genera were also closer in terms of cooperation and competition.

For the necrotic pulp samples, the core genera in the network were *Leptotrichia, Corynebacterium,* and *Dialister. Leptotrichia* and *Corynebacterium* interacted with more genera, while *Dialister* appeared to be more closely related to other bacteria. In the exudate, *Dialister* appeared to be more important and showed a negative interaction with most bacteria (22/31), with most interactions being statistically significant (*p* < 0.01, 21/31).

## 4 Discussion

ECC is an aggressive form of dental caries in children, which left untreated, can result in rapid and extensive cavitation in teeth (rampant caries) that is painful and costly to treat. Furthermore, it affects mostly children from impoverished background, and thus constitutes a major challenge in public health (Hajishengallis et al., 2017). The patients included in this study all had S-ECC features, a high caries prevalence, and multiple primary teeth with apical periodontitis, so the samples and data were more representative.

Unlike previous studies (Hsiao et al., 2012; Kressirer et al., 2018; de Brito et al., 2020; Ordinola-Zapata et al., 2023), which typically only collected one or two samples from different depths on different teeth, this study obtained samples from all levels on the same tooth without the need of tooth extraction. This approach not only minimized discomfort for the pediatric patients, but also facilitated a more comprehensive understanding of disease progression, starting from the initial stage of intact enamel, to the development of decayed dentin, followed by pulp necrosis, and ultimately leading to apical periodontitis, and resulted in data that more accurately reflects the original characteristics of the disease, enabling a more thorough analysis. The findings revealed that the types of bacteria identified were either similar or identical, with only a small number of unique species found in each layer. Nevertheless, the diversity of bacteria varied between layers, potentially due to the adaptation of the microbiota to different habitats, which is similar to the results of previous histological studies (Demant et al., 2021). Bacteria on the tooth surface have a greater opportunity to acquire nutrients and oxygen from food compared to those in deeper layers, leading to higher α-diversity. As the depth increases during caries progression, the environment becomes increasingly anoxic or even anaerobic, resulting in decreased bacterial diversity. For instance, bacteria in the root canal face limited access to nutrients and oxygen and are challenged by the apical immune system, leading to lower microflora diversity. Our analysis of data supports the hypothesis that disease processes contribute to decreases in bacterial diversity. Furthermore, our study illustrated that the bacterial composition of dentin caries falls between that of the tooth surface and pulp canal, suggesting potential migration of bacteria from the surface to the pulp canal as the disease advances. With disease progression, the number of shared bacteria gradually increases, resulting in higher similarity in the microbiota composition. It is also plausible that different bacteria play varying roles at different stages of the disease.

We then proceeded to analyze the dynamics of core taxa at various stages of disease progression, with the initial focus on *Streptococci* and *Lactobacilli*, due to severely deep stages of caries in children having been confirmed in previous studies. Having been confirmed in previous studies (Richards et al., 2017; Xiao et al., 2018a). Our results confirmed that these two genera were very abundant on tooth surfaces and in dentin caries, and as disease progressed, the composition of core taxa within the microbiota also changed, indicating potential alterations in both the structure and function of the microbiota (Banerjee et al., 2018).

We also observed a high relative abundance of *Prevotella* and *Veillonella* in superficial and deep dentin caries layers, indicating that these two genera may also have a significant role in caries progression from outer to inner dentin. Previous studies have shown that *Veillonella* is found commonly in S-ECC lesions and has been linked to the rapid expansion of lesions into the dentin (Liu et al., 2011). Although *Veillonella* does not produce acid, it utilizes lactic acid produced by other acid-producing bacteria as a carbon source. This may support its growth and survival (Liu et al., 2011), potentially allowing it to benefit from the acidic environment of carious lesions (Do et al., 2015). Therefore, *Veillonella* might also be considered as a biomarker of caries progression in ECC patients. The presence of weak or non-acid-producing but proteolytic *Prevotella* in plaque from children with S-ECC has also been associated with progression of dental caries to the dentin level, as this requires denaturation of proteins (Chalmers et al., 2015). Consistent with previous studies (Liu et al., 2020), we also showed *Leptotrichia* were more prevalent in superficial dentin lesions, with these non-motile facultative bacteria having a high potential for saccharolysis and cariogenesis and ability to ferment mono-and disaccharides and produce lactic acid (Eribe and Olsen, 2017).

The microbial composition can vary depending on the different sites of the tooth surface. These microorganisms interact with each other in a dynamic and concerted polymicrobial synergy to form a cariogenic biofilm (that is, a biofilm that can cause caries) within which the community changes as caries progress from early onset (initial demineralization) to deeper lesions with dentin exposure (Bowen et al., 2018).

Our data showed that differences in abundance between genera in the necrotic pulp and exudate were no longer significant. We therefore studied how interactions between these dynamic bacteria evolved with disease development and showed that connections between bacteria became stronger as disease advanced. The functions of the microbiota also became more complex and the core genera in the networks changed as disease progressed. However, these core genera did not align with their relative abundance in the samples. Generally, a negative interaction results from parasitism, predation, amensalism, and competition (Faust and Raes, 2012). Our results indicated that *Lactobacillus* and bacteria such as *Corynebacterium*, *Actinomyces*, and *Olsenella* have significant mutual inhibition and a competitive relationship in dentin caries.

Some studies have reported a link between *Capnocytophaga* and active dental caries (Muhoozi et al., 2022), while others reported a higher abundance of *Capnocytophaga* in children without caries compared to those with active caries, which was defined as cavitated lesions as well as white spot lesions with an opaque, chalky surface (Schoilew et al., 2019). Previous research on *Fusobacterium* also suggested a connection to caries-free states (Schoilew et al., 2019). Our results showed a decrease in the abundance of both *Capnocytophaga* and *Fusobacterium* as caries progressed. However, we also observed a positive correlation between these two genera and other bacteria in superficial and deep dentin lesions (Figure 5), indicating a synergistic effect that could potentially impact caries development.

*Dialister* species are commonly present in the oral cavity, and was reported to be one of the most prominent genera in the root canals of apical periodontitis (Buonavoglia et al., 2023). In our study, *Dialister* was shown to be the core bacteria in a number of network interactions and also within the microflora network in necrotic pulp and exudate samples. This indicates that *Dialister* may play a significant role in necrotic pulp and periapical inflammation.

The changes in core bacteria found in this study during disease progression predict changes in bacterial community function at different disease stages. The collective function of microbial communities is a major driver of homeostasis or dysbiosis and ultimately health or disease (Richard et al., 2018).

The whole is much more than the simple sum of its parts, since the interactions between different parts resulted in many new physiological functions which cannot be observed with individual components. Correlation-based network analysis has been successfully used to explore the co-occurrence patterns of microbial communities (Lyu et al., 2021; Pei et al., 2023), and future work is needed to better understand the role of these keystone bacteria in this study in co-occurrence networks.

The current study had some limitations and drawbacks. Although the 16S rRNA analysis technique had a relatively low cost and allowed for large-scale studies of thousands of samples, bacterial resolution was usually limited to the genera or species level (Human Microbiome Project Consortium, 2012). DNA-sequencing-based approaches cannot generally distinguish between living and metabolically active, damaged, or dead bacterial cells, or free DNA, whereas RNA sequencing analyses identify living and metabolically active cells and also those that are dying (Tamburini et al., 2016). This is an important distinction because the human innate immune system selectively responds to microbial activity (Sander et al., 2011). In addition, studies have shown that there is a strong correlation between intraoral Candida and the onset of S-ECC (Raja et al., 2010; Qiu et al., 2015), and that children with oral *Candida albicans* are five times more likely to develop ECC than children without this infection (Xiao et al., 2018b). Due to the limitations of 16S rRNA technology, our study was unable to investigate the distribution of Candida in different areas. So in the next, these results will be further tested using different molecular methods, such as metagenomics and metatranscriptomics, a larger sample size and so forth.

It is important to note that although we treated and collected samples in separate operating rooms and used rubber dams to isolate the teeth, followed by rinsing with sterile saline before sampling at different levels, there remained a risk of contamination during the sampling process.

More accurate and comprehensive bacterial analyses could be used to improve the accuracy of the existing ECC-risk screening methods, and provide greater predictability of ECC development. This approach once validated could lead to implementation of targeted early intervention and enhanced preventive care for the susceptible children, such as the use of probiotics (La Rosa and Pedullà, 2023; Luo et al., 2024; Yu et al., 2024), thus reducing the economic burdens and painful consequences of the progressed stages of the disease.

Based on a limited sample, our preliminary results are promising. However, to validate these findings, a larger sample is needed. Hence, we advise caution in interpreting these results until further research is conducted.

## 5 Conclusion

In conclusion, the aim of this study was to investigate the profiles of bacterial diversity during progression of S-ECC, within the same tooth, from the tooth surface to the root canal. We observed an increase in both the quantity and complexity of bacterial interactions. This change made the bacterial community more mature and stable, so that it was better able to resist the host immune response and medicaments, and maintained the state of the disease, promoting the progression of the disease. This finding emphasizes the importance of not only examining changes in bacterial abundance, but also considering the interrelationships between bacteria and other microorganisms in the microbial community when investigating the role of bacteria in disease progression. Future studies with larger sample sizes and metatranscriptomic and metabolomics approaches are needed to determine potential pathways that are relevant to promoting caries or metabolism that are inhibitory to caries.

## Data availability statement

The raw reads were deposited into the NCBI Sequence Read Archive (SRA) database https://www.ncbi.nlm.nih.gov/sra, accession number PRJNA1129318.

## Ethics statement

The studies involving humans were approved by Ethical Committee of Peking University School and Hospital of Stomatology (Beijing, China) (approval number: PKUSSIRB-202281143). The studies were conducted in accordance with the local legislation and institutional requirements. Written informed consent for participation in this study was provided by the participants’ legal guardians/next of kin.

## Author contributions

BL: Data curation, Methodology, Writing – original draft, Writing – review & editing, Conceptualization, Formal analysis, Investigation, Resources. JW: Methodology, Validation, Writing – review & editing, Funding acquisition, Supervision. YZ: Data curation, Methodology, Project administration, Supervision, Validation, Writing – review & editing.

## Footnotes

^1^ http://huttenhower.sph.harvard.edu/LEfSe

^2^ http://www.arb-silva.de
